# A Retrospective Study of Unicompartmental Knee Arthroplasty Functional Outcome and the Incidence of Medial Meniscus Posterior Root Tear in Spontaneous Osteonecrosis of the Knee

**DOI:** 10.1155/2021/6614122

**Published:** 2021-04-30

**Authors:** Po-Ju Wu, Tsung Yu Lin, Yung Chang Lu

**Affiliations:** ^1^Department of Orthopedics Surgery, Mackay Memorial Hospital, Taipei City, Taiwan; ^2^Mackay Junior College of Medicine, Nursing and Management, New Taipei City, Taiwan; ^3^Biomechanics Research Laboratory, Department of Medical Research, Mackay Memorial Hospital, New Taipei City, Taiwan; ^4^Mackay Medical College, New Taipei City, Taiwan

## Abstract

**Background:**

Spontaneous osteonecrosis of the knee (SONK) can lead to severe knee osteoarthritis predominantly localized to the medial compartment. We aimed to determine whether unicompartmental knee arthroplasty was an effective treatment for primary SONK.

**Methods:**

We analyzed the functional outcomes in 23 patients with SONK (with a magnetic resonance imaging- (MRI-) confirmed diagnosis) who underwent UKA at a single center. The mean follow-up time was 67 months post-UKA.

**Results:**

Significant improvements in function were indicated by reduced Oxford Knee and Visual Analogue Scale scores after UKA, and there were no specific complications after the procedures. The incidence of MRI-identified medial meniscus posterior root tear (MMPRT) was 69.6% (16/23).

**Conclusion:**

Unicompartmental arthroplasty for SONK is less destructive to the native knee structure than total knee arthroplasty but can achieve comparable prognosis with strict patient selection. While the precise etiology of SONK is unknown, one theory posits that a MMPRT may change the biomechanical circumstances of the knee joint, leading to osteonecrosis. Although not confirmatory, the high prevalence rate suggests that MMPRT may have a key role in the development of SONK. UKA is an effective treatment option for SONK, resulting in significant functional improvement. Long-term (>10 years) outcomes should be investigated.

## 1. Background

Osteonecrosis of the knee is caused by diverse etiologies [1]. The condition can be divided into three subtypes: spontaneous, secondary, and postarthroscopic. Postarthroscopic osteonecrosis (ON) is the least common form, which may occur after menisectomy, cartilage debridement, and radiofrequency surgery. Secondary osteonecrosis usually affects patients below 45 years of age, and it can be secondary to systemic diseases, corticosteroid use, radiation, alcohol abuse, and chemotherapy [2, 3]. Spontaneous osteonecrosis of the knee (SONK) may be induced by microfractures of the subchondral bone whereby it can lead to joint space narrowing or end-stage knee arthritis [4]. SONK is the most common form of osteonecrosis, with the highest prevalence in women and people aged over 60 years. The condition is usually unilateral and predominantly localized to the medial compartment [5].

Treatment options for SONK are based on specific symptoms and the disease stage. Nonsurgical treatments for precollapse SONK include protected weight bearing and analgesic usage as needed [6]. Hyperbaric oxygen therapy may provide a noninvasive treatment by improving oxygenation and reperfusion of ischemic area, but the therapeutic effects in SONK should be further investigated [7]. Surgical treatments included joint-preserving procedures and arthroplasty.

Unicompartmental knee arthroplasty (UKA) is considered as a less destructive treatment option for SONK as it preserves more the native knee and proprioception compared to total knee arthroplasty (TKA) [8]. When osteoarthritis is involved in more than one compartment, TKA is the preferred option.

Advances in both prosthesis design and surgical techniques have led to UKA becoming an increasingly valid surgical option for SONK. However, because of the small sample sizes used in previous studies, it remains controversial whether UKA is comparable to TKA for the treatment of SONK [9–12].

We hypothesized that whether UKA can relieved pain and regain prior functional status in primary SONK patients. Therefore, in this study, we analyzed clinical outcomes of UKA for primary SONK at a mean follow-up period of 67 months. Because it has been proposed that medial meniscus posterior root tear (MMPRT) may play an important part in the development of SONK, we also investigated the incidence of MMPRT in this cohort of SONK patients.

## 2. Methods

This study was conducted in accordance with the Declaration of Helsinki, and the protocol was approved by the Ethics Committee of the MacKay Hospital (Project identification code: 19MMHIS161e). All patients provided informed consent for inclusion in the study.

**Figure 1:**
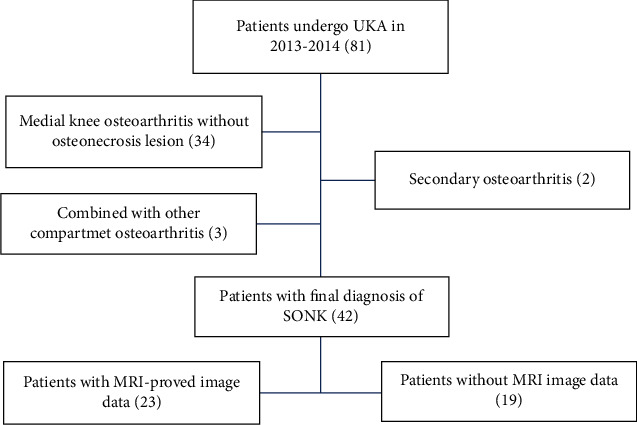
Flow diagram of the patient selection procedure including the exclusion criteria.

**Figure 2:**
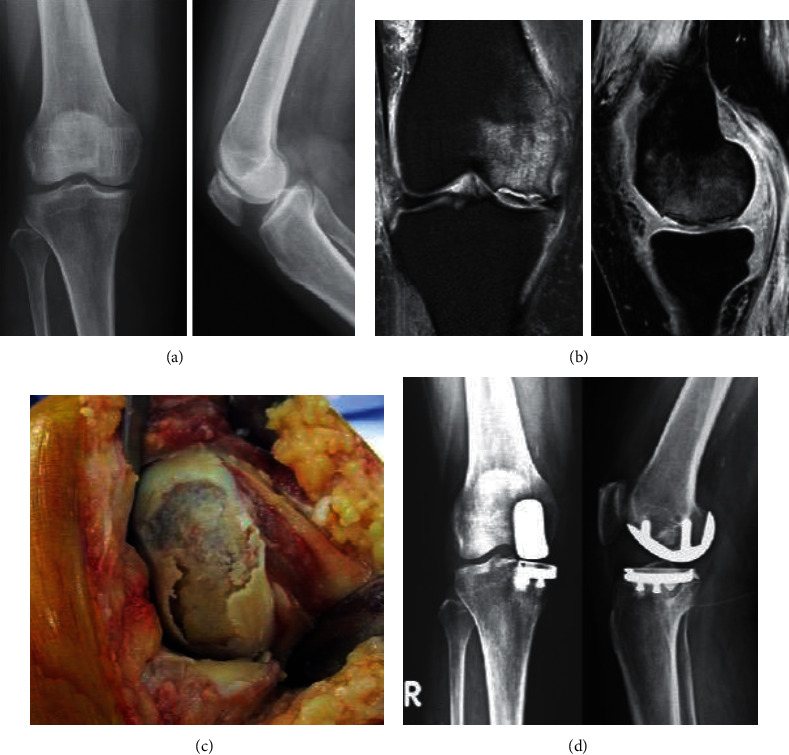
(a) Anteroposterior and lateral knee X-rays showing medial condyle flattening with joint space narrowing. (b) T2-weighted magnetic resonance images showing osteochondral fractures of the medial femoral condyle with marked marrow edema. (c) Clinical picture taken during surgery showing a massive osteochondral fracture in medial femoral condyle. (d) Knee X-rays showing reconstruction status postunicompartmental knee arthroplasty.

We conducted a retrospective chart review of patients who underwent UKA at the Department of Orthopedics, MacKay Hospital (Taipei, Taiwan). We reviewed the charts of all patients (*n* = 81) who underwent UKA using a Physica ZUK (Lima Corporate, Udine, Italy) at our hospital between 2013 and 2014 and followed up for at least 5 years after surgery. The inclusion criterion was patients with primary SONK at the medial femoral condyle of the knee. The exclusion criteria were as follows: (1) single-compartment osteoarthritis without osteonecrosis lesion, (2) secondary osteonecrosis, (3) infection, (4) inflammatory arthritis, (5) flexion contracture, (6) ligament instability, and (7) SONK combined with multiple compartment osteoarthritis (Figure 1). Surgeries were performed in a standard manner with cemented components for all cases. During the procedure, the necrotic bone lesion was removed as possible, with the residual healthy bone serving as the base for cement construction (Figure 2).

During routine care, clinical and radiographic data were collected before and after the operation, and follow-ups were scheduled at 1, 3, and 12 months, respectively, and subsequently once every year. Medical records were reviewed thoroughly to confirm revision, reoperation, and complication rates. The Oxford Knee Score (OKS) [13] and Visual Analogue Scale (VAS) for pain were used to analyze postoperative clinical outcomes. Clinical data for all patients are shown in Table 1. In total, six men and 17 women were included in the study, with an age range of 54–80 years (mean 68.9 years) and body mass index (BMI) range of 21.7–33.2 kg/m^2^ (mean 26.5 kg/m^2^).

Radiographic images were used to diagnose disease stage, ranging from early stage precollapse to late stage subchondral collapse. The Ficat stage was classified using plain X-rays in the A-P view (Table 2).

Preoperative magnetic resonance imaging (MRI) was used to detect meniscal lesions as well as the size and area of necrosis. Necrosis percentage was measured using the condylar ratio in the AP direction, and the volume of SONK was calculated by multiplying the greatest AP width, lateral width, and lesion height in the sagittal view (Table 2).

**Figure 3:**
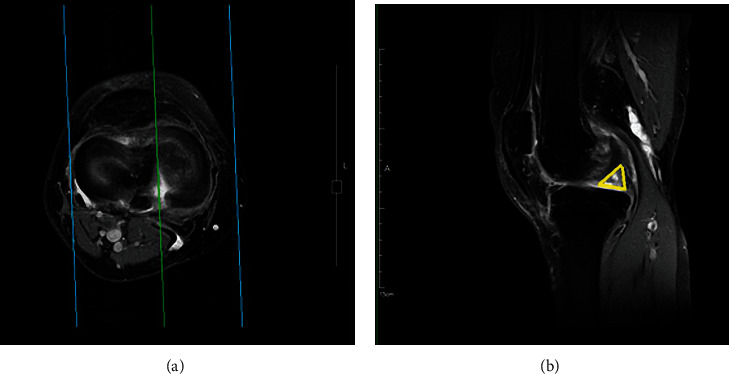
(a) Cleft sign: interruption of the medial meniscus at the posterior horn in axial plane. (b) Ghost sign: empty image of medial meniscus.

Based on a previous report [14], MMPRT was defined by the presence of both following MRI findings:

1. Interruption of the medial meniscus at the posterior horn in the coronal or axial plane, known as the “cleft sign” (Figure 3(a))
2. An empty image of the medial meniscus at the posterior horn in the sagittal plane, known as the “ghost sign” (Figure 3(b))

For the study with continuous outcome variables, the required sample size was calculated with specified power 0.90 and significance level 0.05. According to G∗Power 3.1.9.2, the required sample size was recommended at least 13. All statistical data were analyzed using the SPSS version 22.0 software® (IBM Corporation, Armonk, NY, USA). We conducted the descriptive statistics for SONK patient outcome by pre- and postoperative measurements with OKS and VAS and performed the paired *t*-test for comparing the effects of pre- and postoperative OKS and VAS. The alpha level was set at 0.05, and therefore, the criterion for statistical significance is *p* value <0.05.

## 3. Results

Twenty-three UKA knees due to SONK were included in the final analysis. The mean follow-up period was 67 months (range: 60 to 76 months). There were no severe complications, such as deep vein thrombosis, prosthetic infection, fat embolism, or death in our cohort. MRI findings for all patients are listed in Table 2. The mean condylar ratio was 62.2% (range: 44.0–87.9%) in the AP direction. MRI-confirmed MMPRT was identified in 69.5% (16/23) of all SONK cases.

The mean clinical outcome scores for preop and postop VAS were 8.04 (range: 7.0–9.0) and 2.61 (range: 1.0–4.0), respectively. The mean clinical outcome score for the pre- and postoperative OKS was 16.73 (range: 12.0–32.0) and 40.65 (range: 32.0–48.0), respectively (Table 3). Comparing clinical outcomes post- versus presurgery, the postoperative VAS and OKS were significantly improved than the preoperative scores (both *p* < 0.001) (Table 4).

## 4. Discussion

Unicompartment knee arthroplasty is an effective treatment for SONK patient. In this study, knee pain was alleviated, and the function of the knee improved after UKA. SONK is a common condition that can be treated nonoperatively or with joint-preserving surgical intervention [15, 16]. SONK can progress to articular collapse and end-stage joint osteoarthritis despite medical intervention. Knee arthroplasty for SONK includes both TKA and UKA approaches; however, it remains controversial which approach results in the best surgical outcome for SONK. The indications for UKA are much stricter than those of TKA for patients with osteonecrosis. UKA is utilized in single-compartment osteonecrosis without degenerative changes in other compartments. Intact ligament structures with virtually normal alignment are also required. Several previous studies reported that TKA gave better outcomes than UKA [16, 17]. However, using current surgical techniques, UKA can preserve the anterior cruciate ligament and noninvolved cartilage in other knee compartments, all of which are sacrificed in TKA. As a result, UKA is considered a better option for SONK patients with only single compartment involvement. In previous studies, UKA had a higher risk of revision and worse clinical outcomes than TKA for single compartment osteoarthritis [18, 19]. Using a modern implant design and modified surgical techniques, Myers et al. found that inadequate patient selection and improper surgical indication wound resulted in high revision rates and poor clinical outcomes [9, 20, 21]. Kozinn et al. concluded that UKA was contraindicated in patients with inflammatory arthritis, tricompartmental knee arthritis, fixed varus deformity greater than 10°, fixed valgus deformity greater than 5°, flexion contracture greater than 15°, and knee instability without an intact anterior cruciate ligament [22].

A prospective study of SONK treated with UKA showed significant improvement in OKS postoperatively. UKA appears to be the treatment of choice with the advantage of minimizing bone stock destruction, decreasing the use of bone cement, and better preservation of knee function in comparison to outcomes following TKA [23].

Chalmers et al. found that the success rates of UKA without revision surgery at 5 and 10 years postoperatively were 89% and 76%, respectively. Three cases (7.1%) were converted to TKA, one for development of lateral osteonecrosis (secondary osteonecrosis due to steroid treatment) and two for lateral compartment degeneration [24]. Secondary osteonecrosis of the knee can be caused by alcohol abuse, high-dose systemic corticosteroid use, or other direct cause [2]. Misdiagnosed secondary osteonecrosis was a risk factor for UKA success and further revision surgery.

Polyethylene wear is another issue, and some studies reported that fixed-bearing UKA may produce substantial wear due to malposition. The incidence of polyethylene wear is rare in mobile-bearing UKA because this prosthesis preserves more of the natural kinematics of the knee. However, all of our cases were conducted with fixed-bearing prostheses, and none showed obvious signs of wear on follow-up radiographic images [25].

Because the precise etiology of SONK is currently unknown, effective management of the condition remains challenging for orthopedists. In the past, SONK was thought to result from focal ischemia that caused bone necrosis. Another hypothesis was that SONK may be caused by subchondral insufficiency fractures. Subchondral insufficiency fractures may produce fluid accumulation in the bone marrow, bringing on focal ischemia and ultimately necrosis [4, 26]. Subchondral insufficiency fractures may be attributed to pathological lesions, including osteoporosis, meniscus tear, or meniscectomy.

A recent study in support of this alternative hypothesis demonstrated that meniscus tears are related to cartilage destruction in the knee joint. Allaire et al. proposed that MMPRT may alter the knee's natural biomechanics and increase the peak contact pressure in the joint. The authors even raised the possibility that the effect after MMPRT injury was comparable to the patients' status posttotal medial meniscectomy [27]. The incidence of MRI-identified MMPRT was 69.6% (16/23 cases) in our study cohort. A retrospective analysis by Robertson et al. found a similar high incidence of MRI-identified MMPRT (80% of patients) in SONK. They proposed that femoral overload with increasing interosseous pressure by meniscal discontinuity is one of the causes of SONK [28]. In comparison to patients with knee osteoarthritis, the incidence of MMPRT and the level of posterior tibia slope were higher in SONK patients. Yamagami et al. proposed that MMPRT and higher posterior tibia slope have a greater association with SONK development [14]. Although the definite mechanism remains unknown, many studies have proposed that MMPRT has a key role in the development of SONK.

This study has limitations. First, we utilized a retrospective design with a small number of patients. Second, only short-term follow-up was available. No long-term radiographic outcomes or complication rates from polyethylene wear or implant loosening were available for assessment. Future long-term prospective studies are needed to fully understand the functional outcomes and complication rates.

## 5. Conclusions

UKA is an effective treatment option for SONK, resulting in significant functional improvement and pain relief. Long-term outcomes need to be investigated. A high incidence of MMPRT was noted in our patient cohort, suggesting that the relationship between SONK and MMPRT requires further research.

## Figures and Tables

**Table 1:** Clinical data of all patients.

Case no.	Sex	Age (years)	BMI (kg/m^2^)	Follow-up (months)
1	F	71	30.1	76
2	M	68	26.6	74
3	F	62	28.5	74
4	F	60	26.7	72
5	F	75	28.1	71
6	F	80	26.3	71
7	F	77	24.9	70
8	M	60	27.7	69
9	M	72	22.8	69
10	F	68	24.3	69
11	F	78	25.1	68
12	M	64	23.7	67
13	F	69	21.7	67
14	F	73	23.9	66
15	F	54	22.3	65
16	F	67	23.3	65
17	F	69	36	64
18	F	64	30.5	64
19	F	71	25.4	62
20	M	70	28	62
21	F	77	24.4	61
22	M	77	25.2	60
23	F	59	33.2	60

**Table 2:** Radiographic data.

Case no.	Ficat stage	Condylar ratio (%)	SONK volume (cm^3^)	Meniscus tear in MRI
1	II	61.4	5.833	MMPRT (+)
2	II	67.4	4.118	MMPRT (+)
3	II	44.0	1.349	MMPRT (+)
4	III	62.7	4.147	MMPRT (+)
5	III	87.9	6.881	MMPRT (+)
6	II	63.1	5.017	MMPRT (+)
7	III	67.2	4.672	MMPRT (+)
8	III	53.2	7.977	(-)
9	I	54.8	2.786	(-)
10	I	65.4	14.114	MMPRT (+)
11	III	68.3	12.41	MMPRT (+)
12	II	48.0	1.854	Medial meniscus anterior horn tear, MMPRT (-)
13	II	61.5	5.707	MMPRT (+)
14	I	56.6	10.731	Medial meniscus tear
15	I	62.4	9.309	MMPRT (+)
16	III	59.5	10.731	MMPRT (+)
17	III	65.6	4.146	MMPRT (+)
18	II	71.2	20.694	MMPRT (+)
19	III	63.9	11.727	Medial meniscus anterior horn tear, MMPRT (-)
20	II	52.5	2.829	Medial meniscus anterior horn tear, MMPRT (-)
21	III	66.7	9.108	MMPRT (+)
22	II	48.8	7.749	(-)
23	II	78.9	18.022	MMPRT (+)
		Mean: 62.2%		MMPRT (+): 16/23 cases

SONK: spontaneous osteonecrosis of the knee; MMPRT: medial meniscus posterior root tear.

**Table 3:** SONK patient outcomes.

	Minimum	Maximum	Mean ± std.
Preop VAS	7.0	9.0	8.04 ± 0.77
Postop VAS	1.0	4.0	2.61 ± 0.72
Preop OKS	12	32	16.73 ± 5.06
Postop OKS	32	48	40.65 ± 4.28

SONK: spontaneous osteonecrosis of the knee; VAS: Visual Analogue Scale; OKS: Oxford Knee Score.

**Table 4:** Statistical comparison of pre- and postoperative outcome scores.

Preoperative	Postoperative	95% CI	*T*	*p* value
Preop VAS	Postop VAS	(5.0700, 5.7995)	30.901	<0.001
Preop OKS	Postop OKS	(21.2991, 26.5269)	18.973	<0.001

VAS: Visual Analogue Scale; OKS: Oxford Knee Score.

## Data Availability

The authors confirm that the data supporting the findings of this study are available within the article.

## Abbreviations

SONK: Spontaneous osteonecrosis of the knee
UKA: Unicompartmental knee arthroplasty
TKA: Total knee arthroplasty
MMPRT: Medial meniscus posterior root tear
VAS: Visual Analogue Scale
OKS: Oxford Knee Score
MRI: Magnetic resonance imaging.
